# Sunscreen Use and Photoprotection Practices: A Cross-Sectional Survey of Adults in a Dermatology Outpatient Clinic

**DOI:** 10.7759/cureus.84783

**Published:** 2025-05-25

**Authors:** Asli Tatliparmak

**Affiliations:** 1 Department of Dermatology, Memorial Bahçelievler Hospital, Istanbul, TUR; 2 Department of Dermatology, Uskudar University, Istanbul, TUR

**Keywords:** health behavior, machine learning, skin neoplasms prevention & control, sunlight, sunscreening agents

## Abstract

Background

Ultraviolet (UV) radiation is a leading environmental cause of photoaging and skin cancer. Despite widespread public health recommendations, sunscreen use remains inconsistent in many populations.

Methods

We conducted a cross-sectional survey of 200 adults at a dermatology outpatient clinic in Istanbul, Turkey. The Turkish-language questionnaire assessed sunscreen habits, beliefs, UV risk awareness, and complementary sun protection behaviors. Behavioral profiles and predictors of use were analyzed using multivariate methods.

Results

Sunscreen use was reported by 72.5% of participants, but only 27.0% used it daily and 27.5% never used it. Most applied it only in the morning (51.5%), and 23.0% reported reapplication. Full awareness of sun damage and belief in sunscreen's cancer prevention were low (23.5% and 18.0%, respectively). The mean adherence score was 3.0/5. Common alternative protections included sunglasses (60.0%) and hats (46.0%). Non-use was mainly due to forgetfulness, perceived lack of need, and cost. Clustering revealed three behavior profiles; one group showed high awareness and consistent use. A machine learning model explained 2.5% of adherence variance; key predictors were age, cancer prevention motivation, and hat use.

Conclusion

Sunscreen behavior in this outpatient sample revealed inconsistencies between self-reported use and recommended practices. The findings highlight the need for further research and targeted public health strategies to improve awareness and application habits.

## Introduction

Ultraviolet (UV) radiation is a well-established environmental factor implicated in photoaging, pigmentary disorders, and various cutaneous malignancies, including both melanoma and non-melanoma skin cancers. UV radiation damages DNA, lipids, and proteins in skin cells, triggering complex biological responses and increasing the risk of carcinogenesis [1]. Numerous studies have confirmed the direct role of UV exposure in the development of skin cancers, supporting robust public health efforts focused on photoprotection [2]. Although preventive strategies, such as broad-spectrum sunscreen use, wearing protective clothing, and limiting midday sun exposure, are widely recommended, adherence remains persistently suboptimal [3]. Recent national and international surveys consistently show that fewer than half of adults use sunscreen regularly, even when exposed to high UV indices [4].

Understanding sun protection behavior requires a multidimensional approach encompassing cognitive, behavioral, and contextual factors. The Health Belief Model (HBM) and the Theory of Planned Behavior (TPB) have been instrumental in explaining how perceived susceptibility, perceived benefits and barriers, and behavioral control shape individuals' decisions about photoprotection [5,6]. Empirical evidence further highlights the predictive power of these frameworks in various populations, including caregivers, farmers, and adolescents [7,8]. Structured surveys remain the most practical tools to assess such constructs at the population level and are essential for capturing behavioral heterogeneity and evaluating public health interventions [9]. While self-reported behaviors can be affected by recall bias or social desirability effects, culturally adapted and anonymous survey instruments have shown validity in identifying meaningful behavioral phenotypes [10].

This study aimed to investigate sunscreen-related behaviors in a community-based adult population without known dermatologic diagnoses, focusing on the general population's baseline attitudes and practices. The instrument, developed in Turkish and culturally adapted, was pilot-tested for psychometric consistency. The survey covered domains such as sunscreen use frequency, perceived efficacy, reapplication habits, and use of complementary photoprotection strategies. Advanced analytics, including unsupervised clustering (with silhouette coefficient validation) and gradient-boosted modeling, were applied to identify behavioral phenotypes and the most influential predictors of sunscreen usage.

## Materials and methods

Study design and setting

This single-center, cross-sectional observational study was conducted at the dermatology outpatient clinic of a university-affiliated tertiary hospital in Istanbul, Turkey, between January and March 2024. The setting provides ambulatory dermatologic care to a diverse population with varying educational and socioeconomic backgrounds. This study aimed to investigate sunscreen-related behaviors in a general adult outpatient population, with data collected prior to dermatologic evaluation and regardless of diagnosis.

Participants and recruitment

A consecutive, non-selective recruitment strategy was implemented to minimize sampling bias. During the two-month study period (January-March 2024), all adult patients (≥18 years) presenting to the dermatology outpatient clinic were approached on weekdays during standard outpatient hours (09:00-16:00). A trained research assistant was present in the waiting area and systematically invited each patient before their clinical consultation. Patients were informed that participation was voluntary and anonymous. Those who agreed provided written informed consent and completed the questionnaire prior to being seen by a dermatologist. Due to logistical constraints, such as high patient volume or simultaneous clinic flow, not every eligible individual could be approached. However, we attempted to minimize missed opportunities by being present continuously and applying the same recruitment procedure throughout the study period. Patients were excluded only if they explicitly declined or were unable to provide informed consent. No exclusions were made based on complaint, clinical suspicion, or sociodemographic characteristics.

Survey instrument development

The structured questionnaire was developed de novo in Turkish, informed by domains commonly used in prior research on sunscreen use, consumer photoprotection behavior, and dermatologic health literacy. The instrument comprised 20 items covering demographic characteristics, sunscreen usage patterns, motivations for use, beliefs regarding sunscreen efficacy, and awareness of UV-related risks. Additional items assessed the use of complementary physical photoprotective measures and, where applicable, sun protection practices for children. Most items were categorical, with some allowing multiple responses. Awareness and belief questions used four-point ordinal scales; adherence was self-rated on a five-point Likert scale.

Although the instrument was not formally validated, its content was structured to reflect key domains identified in prior studies of sunscreen use and photoprotection behavior, with careful attention to conceptual clarity and plain language [5,6]. This expert review was informal and aimed at ensuring cultural appropriateness and conceptual coverage. A formal pilot test or cognitive interview process was not conducted, which is acknowledged as a study limitation. The final version of the questionnaire is available in the Appendices.

Survey administration

Participants completed the paper-based survey in the outpatient waiting area prior to their clinical appointment. A research assistant was present to assist with item comprehension, especially for older adults or individuals with limited literacy. The mean completion time was 8.3 minutes (range: 7-10). Upon submission, responses were reviewed for missing data, and participants were asked to clarify incomplete answers when possible. Contradictory responses, such as reporting never using sunscreen but indicating high adherence, were flagged and reviewed.

Data entry and management

Completed surveys were stored securely and manually entered into a password-protected electronic database. Two trained research assistants performed data entry independently. A 10% random sample of entries was cross-checked for accuracy, with an error rate of less than 2%; discrepancies were resolved by review of original paper forms. Data were coded and preprocessed in Microsoft Excel (Microsoft Corporation, Redmond, Washington) and R version 4.4.2 (R Foundation for Statistical Computing, Vienna, Austria). Ordinal responses were preserved for analysis, while multi-response categorical items (e.g., reasons for non-use) were converted into binary indicators. No imputation was applied, and only complete cases were included in the downstream analysis.

Ethical considerations

Ethical approval was granted by the Non-Interventional Research Ethics Committee of Üsküdar University (approval date: 28/02/2025; reference no: 61351342/020-898). All participants provided written informed consent before participation. No personal identifiers were collected, and all data were anonymized prior to analysis.

Analysis

All statistical analyses were conducted using R version 4.4.2. Descriptive statistics were used to summarize participant demographics and behavioral characteristics. Categorical variables were expressed as counts and percentages, and continuous variables as means with ranges or standard deviations, as appropriate. Unsupervised clustering was performed using k-means on scaled variables related to sunscreen-related behavior, including awareness, belief in efficacy, usage frequency, reapplication habit, self-rating, and physical protection methods (hat, sunglasses, long sleeves). The optimal number of clusters was determined by the elbow method and validated using silhouette scores. To predict participants' self-rated sunscreen usage habits (on a 1-5 scale), a supervised machine learning model was developed using the XGBoost regression algorithm. The model was trained on 89 participants and tested on 38, using early stopping with a patience of 10 rounds to reduce overfitting. The optimal number of boosting iterations was determined as 11. Model performance was evaluated using root mean squared error (RMSE) and coefficient of determination (R²). Feature importance was assessed using SHAP (SHapley Additive exPlanations) values. SHAP summary plots were generated to identify the most influential predictors. Additionally, SHAP interaction values were computed and visualized as a heatmap to explore pairwise interactions among the top five contributing features.

## Results

A total of 200 adult patients were included in the study. The mean age was 42.7 years (range: 18-85), and 58% of participants were female. Regarding educational background, 43.5% had a university degree, 29.5% completed high school, 16.5% had a middle school education, and 10.5% had completed primary school. Sunscreen use was reported by 72.5% of participants. Among all participants, 27.0% reported using sunscreen daily, 22.0% only when it is sunny, and 23.5% only during summer. Meanwhile, 27.5% stated that they never use sunscreen. In terms of awareness of the harmful effects of sun exposure, 23.5% of participants reported being fully aware, 37.0% quite aware, 33.5% slightly aware, and 6.0% not aware at all. When asked about their belief in sunscreen's ability to prevent skin cancer, 18.0% fully believed in its protective effect, 26.0% believed quite a bit, 42.5% somewhat, and 13.5% did not believe at all.

A total of 43.5% of participants reported having used sunscreen based on a dermatologist's recommendation. When asked about the most important factor when choosing a sunscreen, 26.0% indicated SPF level, 17.5% dermatologist recommendation, 13.5% price, 8.5% brand, and 7.0% other; 27.5% did not provide a selection (typically non-users). With regard to sunscreen application habits, 51.5% applied sunscreen only in the morning, 23.0% reapplied during the day, and 25.5% reapplied only when it was very sunny. The average self-reported sunscreen habit score was 3.0 (out of 5). When asked about alternative sun protection methods on sunny days, 60.0% of participants reported using sunglasses, 46.0% wore hats, 32.0% wore long-sleeved clothing, while 14.0% did not use any additional protection. Among those who reported using sunscreen, 47.5% stated that their primary motivation was preventing skin cancer, 39.5% for preventing pigmentation spots, 34.0% to avoid premature aging, and 5.0% for other reasons.

Among the 55 non-users (27.5%), the most common reasons for not using sunscreen were forgetting to apply it (14.0%), perceiving it as unnecessary (9.5%), price concerns (7.0%), and difficulty finding a suitable product (3.5%). Of all participants, 63.0% reported having children. Among these, 74.6% stated that they apply sunscreen on their children. When choosing a sunscreen for their children, 67.5% prioritized SPF level, 49.2% preferred hypoallergenic products, 42.1% followed dermatologist recommendations, and 7.9% considered other factors.

**Figure 1:**
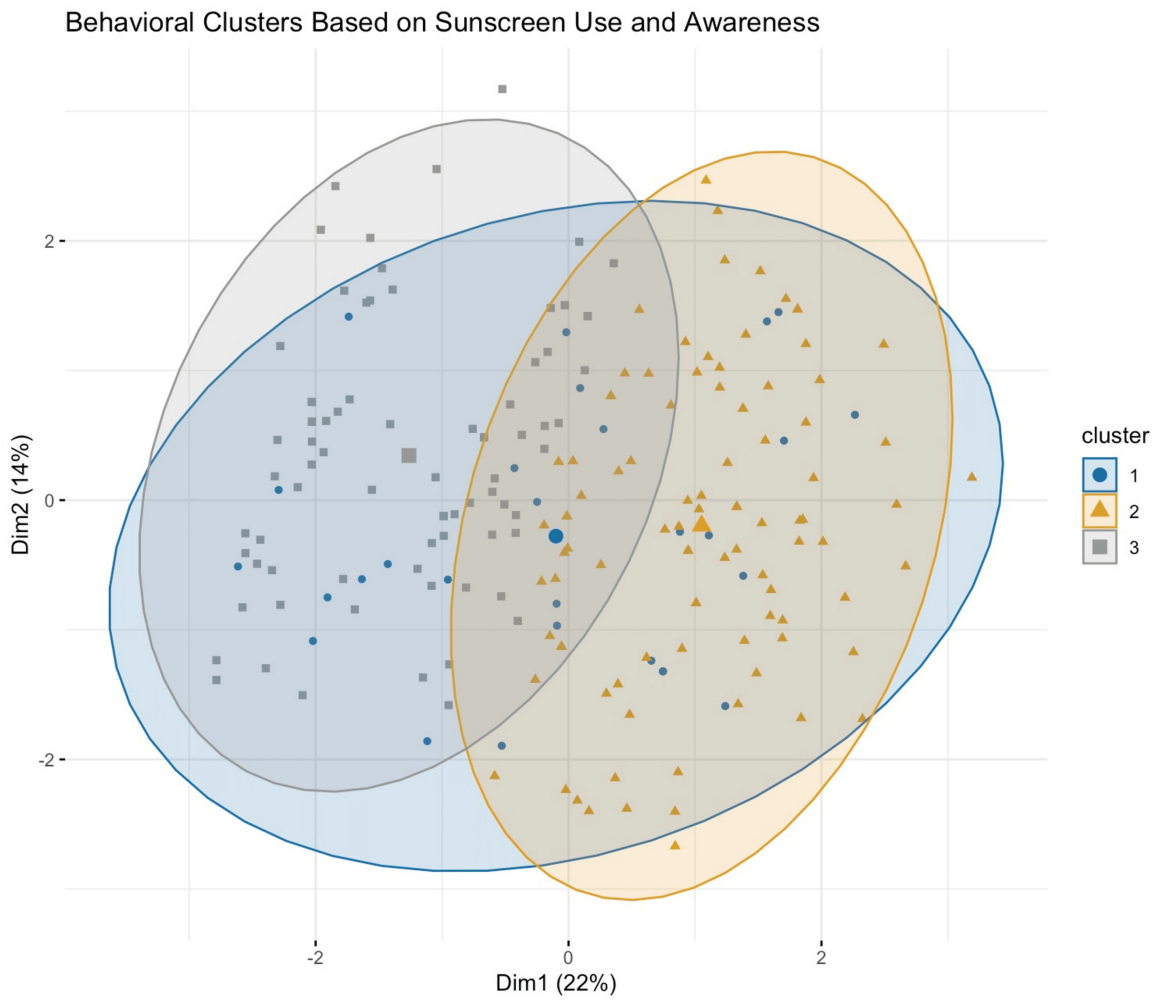
Principal component analysis (PCA) plot showing behavioral clustering of participants based on sunscreen-related variables Cluster 1 (blue circles) represents participants with moderate awareness and moderate sunscreen adherence; Cluster 2 (orange triangles) includes individuals with low awareness and lower adherence despite similar self-ratings; and Cluster 3 (gray squares) reflects participants with high awareness, a strong belief in sunscreen efficacy, and consistent sunscreen use and reapplication. Dim1: First principal component (Dimension 1) explaining 22% of the total variance; primarily reflects differences in sunscreen adherence and belief in effectiveness. Dim2: Second principal component (Dimension 2) explaining 14% of the total variance; captures variation in awareness and knowledge related to sun protection.

K-means clustering was performed on scaled behavioral data, including awareness, belief in sunscreen efficacy, usage frequency, reapplication habit, self-rated behavior, and physical sun protection methods (Figure 1). The optimal number of clusters was determined to be three based on the elbow method and internal validation. The mean silhouette score was 0.161, indicating modest internal separation. Cluster 1 (n = 28) included participants with intermediate awareness and belief scores (2.82 and 2.46, respectively), relatively lower self-ratings (mean = 2.82), and modest behavior metrics (Table 1). Only 21.4% in this group reported daily sunscreen use, and 17.9% reported reapplying during the day. Cluster 2 (n = 95) was characterized by the lowest awareness (mean = 2.17) and belief scores (1.74), suggesting lower engagement with sun protection. Although their self-rating was similar to Cluster 1 (2.91), their actual behavior scores were slightly higher than Cluster 1 in daily use and reapplication. This group had the lowest proportion of hat use (42.1%). Cluster 3 (n = 77) showed the highest scores across awareness (3.52), belief (3.42), and self-rating (3.08). They also had the highest rates of daily sunscreen use (31.2%) and daytime reapplication (26.0%). This group likely represents participants with consistent and informed sun protection behavior. These clusters reveal distinct behavior profiles ranging from high-engagement individuals to those with lower awareness and belief, despite similar self-assessments.

**Table 1:** Behavioral characteristics by cluster

Characteristic	Cluster 1 (n = 28)	Cluster 2 (n = 95)	Cluster 3 (n = 77)
Self-rating, mean (1–5)	2.82	2.91	3.08
Awareness score (1–4)	2.82	2.17	3.52
Belief score (1–4)	2.46	1.74	3.42
Daily sunscreen use	6 (21.4%)	24 (25.3%)	24 (31.2%)
Reapplies during the day	5 (17.9%)	21 (22.1%)	20 (26.0%)
Uses sunglasses	17 (60.7%)	56 (58.9%)	47 (61.0%)
Uses hat	15 (53.6%)	40 (42.1%)	37 (48.1%)

**Figure 2:**
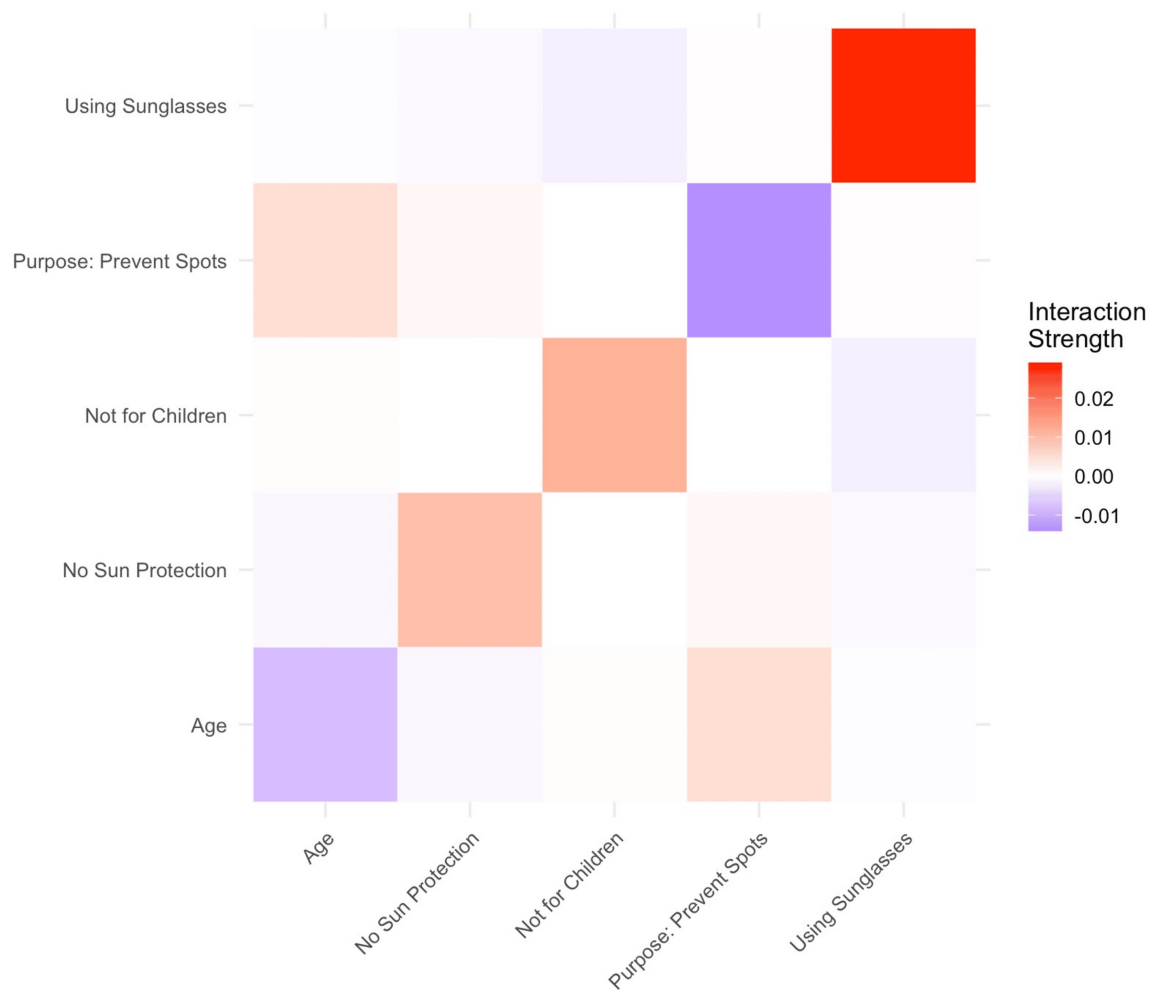
Pairwise SHAP interaction heatmap of the top five predictors of sunscreen usage habits SHAP: SHapley Additive exPlanations

A supervised machine learning model was built using XGBoost regression to predict participants' self-rated sunscreen usage habits (scale: 1 to 5). The model was trained with 89 observations and tested on 38, using early stopping after 10 rounds to prevent overfitting. The best iteration occurred at round 11. The model's performance on the validation set yielded an RMSE of 1.059 and an R² of 0.025, indicating that 2.5% of the variance in self-rated behavior was explained by the model. SHAP values were calculated to assess feature importance. The top three predictors were age (SHAP importance: 0.207), using sunscreen to prevent skin cancer (0.176), and wearing a hat on sunny days (0.099) (Table 2). Additional influential variables included belief in sunscreen effectiveness (cubic-transformed), not using sunscreen for children, using sunscreen to prevent pigmentation spots, wearing sunglasses, and reapplying only in the morning. To explore how these factors interact, a SHAP interaction heatmap was generated based on the top five most influential variables. The plot displays pairwise interaction strengths between features such as age, sunglasses use, no additional sun protection, use for preventing pigmentation, and not applying sunscreen to children (Figure 2). Stronger interactions were observed between sunglasses use and no protection and between not using sunscreen for children and spot prevention. These interaction values are averaged across all observations in the test set.

**Table 2:** SHAP feature importance for predicting self-rated sunscreen usage habits SHAP: SHapley Additive exPlanations

Rank	Feature	SHAP Importance
1	Age	0.207
2	Purpose: cancer prevention	0.176
3	Using hat	0.099
4	Belief in sunscreen effectiveness (modeled as a cubic term)	0.095
5	Not using sunscreen for children	0.089
6	Purpose: preventing spots	0.085
7	Using sunglasses	0.080
8	Reapply (only morning)	0.050
9	Wearing long sleeves	0.049
10	Education level (modeled as a cubic term)	0.045

## Discussion

This study shows that although 72.5% of participants reported using sunscreen, only 27.0% applied it daily and 27.5% never used it. Awareness of sun-related harm and belief in sunscreen efficacy were low, with fewer than one in four expressing strong agreement in either domain. Self-reported adherence did not always align with recommended practices, particularly regarding reapplication. Behavioral drivers such as age, motivation to prevent skin cancer, and physical sun protection habits emerged as key influences on sunscreen use.

A closer examination of application habits further highlights the disconnect between perceived and actual photoprotection. In this study, 51.5% of participants reported applying sunscreen only once in the morning, and just 23.0% reported any reapplication throughout the day, despite consistent recommendations advocating reapplication every two hours, especially after sweating or water exposure [11]. This discrepancy mirrors findings from global surveys, which show that even among regular sunscreen users, adherence to reapplication guidelines remains low, often due to forgetfulness, inconvenience, or misconceptions about product longevity [12,13]. Although participants in our study self-rated their sunscreen behavior moderately (3.0 out of 5), this confidence likely reflects a misunderstanding of what constitutes effective sun protection. This pattern of overestimated adherence has been observed in broader populations and specific at-risk groups, where users equate any sunscreen use with adequate protection, regardless of timing or thoroughness [14,15]. The belief that a single morning application is sufficient may also stem from a false sense of security reinforced by marketing or SPF labeling, despite evidence that real-life application rarely meets laboratory testing standards [16]. Together, these findings support the need for targeted education campaigns that go beyond general awareness and instead focus on critical behavioral details, especially correct application techniques and the necessity of reapplication. By emphasizing these actionable habits rather than just promoting sunscreen in general, public health efforts may more effectively reduce the gap between perceived and actual protection.

Awareness and belief in sunscreen efficacy were limited across the study population. Only 23.5% of participants reported being fully aware of the harmful effects of sun exposure, and just 18.0% fully believed that sunscreen can prevent skin cancer. These findings align with previous research showing widespread deficits in public understanding of UV risk and photoprotection, even in medically literate populations [17,18]. For instance, studies among college students and agricultural workers have similarly revealed high recognition of skin cancer risk but poor sunscreen adherence and frequent overestimation of personal protection practices [19,20]. K-means clustering further contextualized these patterns by identifying three distinct behavioral profiles. Cluster 3, which comprised 77 participants, exhibited the highest levels of awareness (mean = 3.52) and belief (mean = 3.42) and also had the most consistent behavior, with 31.2% reporting daily sunscreen use and 26.0% reporting daytime reapplication. This convergence between awareness, belief, and actual behavior reinforces the critical role of knowledge in motivating protective habits [21,22]. In contrast, Cluster 2 (n = 95) demonstrated the lowest awareness (mean = 2.17) and belief (mean = 1.74) scores, yet had self-rated sunscreen adherence comparable to the other groups. This mismatch between perceived and actual adherence has also been observed in other populations and may be influenced by misperceptions about what constitutes effective protection [23,24]. For example, individuals may consider occasional sunscreen use or using a low-SPF product as sufficient, despite lacking proper application technique or frequency.

The combination of low objective knowledge and inflated self-perception in our findings highlights a broader challenge in public health communication. Informational interventions that solely emphasize the dangers of UV exposure may be insufficient unless they also correct behavioral misconceptions and encourage accurate self-assessment [25,26]. Behavioral clustering, as used in this study, provides a valuable method to tailor messaging for subgroups that may not respond to general awareness campaigns. Targeted interventions focusing on belief accuracy and behavioral precision, such as proper timing, reapplication, and amount, may significantly enhance the effectiveness of public education efforts [27].

Predictive modeling using XGBoost showed limited explanatory power, with an R² of 0.025 and RMSE of 1.059. SHAP analysis identified age (0.207), motivation to prevent skin cancer (0.176), and hat use (0.099) as the top predictors of self-rated sunscreen adherence. However, the model explained only 2.5% of the variance, suggesting key influences, such as habits, personality, and context, were not captured. This aligns with evidence that sunscreen behaviors are often driven by habits and situational cues rather than conscious intention alone [28,29]. Modeling studies also show behavior is better predicted when accounting for dynamic, habitual patterns and environmental triggers or individual personality traits [30]. These results highlight the limitations of relying solely on structured survey responses to explain complex health behaviors and suggest that future predictive models may benefit from integrating real-time, context-sensitive data, such as ecological assessments, behavioral patterns, and psychological traits, to better capture the full scope of sunscreen adherence behavior.

Limitations

This study has several limitations that should be considered when interpreting the findings. First, the survey instrument was not formally validated, and although it was informed by prior frameworks and reviewed for content clarity, psychometric testing was not performed. Second, the cross-sectional design captures behavior and attitudes at a single time point, limiting inference on causality or temporal changes. Third, all data were self-reported, which may introduce recall bias and social desirability effects, particularly for behaviors perceived as favorable. Fourth, while the sample reflects a typical outpatient dermatology population in an urban tertiary center, the findings may not be generalizable to other settings or populations with different health access profiles. Finally, the machine learning model only accounted for a small proportion of variance in self-rated sunscreen adherence, suggesting that important unmeasured factors, such as psychological traits or environmental influences, were not captured. Despite these limitations, the study provides novel insights into sunscreen behavior patterns and highlights key targets for public health intervention.

## Conclusions

This study highlights the complexity of sunscreen-related behavior, revealing a disconnect between perceived and effective photoprotection. While self-reported use was common, adherence to recommended practices was inconsistent and shaped by multiple behavioral dimensions. Clustering and machine learning analyses revealed limited predictive power of standard variables, suggesting the influence of unmeasured factors. Future studies should incorporate additional dimensions such as awareness of UV index, product selection based on individual skin conditions or life circumstances, and perceived short- and long-term benefits of sunscreen use. Such comprehensive frameworks may better inform behaviorally grounded public health interventions.

## Appendices

Sunscreen Behavior Survey Instrument (English Translation)

Sex: ( ) Female ( ) Male

Age: ____ years

Educational Level: ( ) Primary school ( ) Middle school ( ) High school ( ) University or higher

Do you use sunscreen? ( ) Yes ( ) No

How often do you use sunscreen? ( ) Daily ( ) Only on sunny days ( ) Only during summer months ( ) Never

What is your main reason for using sunscreen? (Select all that apply) ( ) To prevent skin cancer ( ) To prevent premature aging ( ) To prevent pigmentation ( ) Other: ________

What is the most important factor when selecting a sunscreen product? ( ) Price ( ) Brand ( ) SPF level ( ) Dermatologist recommendation ( ) Other: ________

How aware are you of the harmful effects of sun exposure on the skin? ( ) Not at all aware ( ) Slightly aware ( ) Quite aware ( ) Fully aware

To what extent do you believe sunscreen protects against skin cancer? ( ) Not at all ( ) Somewhat ( ) Quite a bit ( ) Completely

Have you ever used sunscreen upon a dermatologist’s recommendation? ( ) Yes ( ) No

If you do not use sunscreen, what are your main reasons? (Select all that apply) ( ) Price ( ) Do not see the need ( ) I forget to apply it ( ) Cannot find a suitable product for my skin

How would you rate your sunscreen-related habits on a scale from 1 to 5? (1 = Very poor, 5 = Excellent) ( ) 1 ( ) 2 ( ) 3 ( ) 4 ( ) 5

What other protective measures do you use on sunny days? (Select all that apply) ( ) Hat ( ) Sunglasses ( ) Long-sleeved clothing ( ) None

Do you apply sunscreen only in the morning or do you reapply during the day? ( ) Only in the morning ( ) I reapply during the day ( ) I reapply only when it is very sunny

Do you have children? ( ) Yes ( ) No

If yes, do you apply sunscreen to your children? ( ) Yes ( ) No

What features do you prioritize when choosing sunscreen for your children? (Select all that apply) ( ) SPF level ( ) Hypoallergenic formulation ( ) Dermatologist recommendation ( ) Other: ________
